# Multibeam 3D Underwater SLAM with Probabilistic Registration

**DOI:** 10.3390/s16040560

**Published:** 2016-04-20

**Authors:** Albert Palomer, Pere Ridao, David Ribas

**Affiliations:** Vicorob Research Institute, Universitat de Girona, c/Pic de Peguera 13-Parc Científic i Tecnològic de la UdG-CIRS Building, Girona l17003, Spain; pere@eia.udg.edu (P.R.); dribas@udg.edu (D.R.)

**Keywords:** AUV, multibeam, SLAM, 3D, bathymetry

## Abstract

This paper describes a pose-based underwater 3D Simultaneous Localization and Mapping (SLAM) using a multibeam echosounder to produce high consistency underwater maps. The proposed algorithm compounds swath profiles of the seafloor with dead reckoning localization to build surface patches (*i.e.*, point clouds). An Iterative Closest Point (ICP) with a probabilistic implementation is then used to register the point clouds, taking into account their uncertainties. The registration process is divided in two steps: (1) point-to-point association for coarse registration and (2) point-to-plane association for fine registration. The point clouds of the surfaces to be registered are sub-sampled in order to decrease both the computation time and also the potential of falling into local minima during the registration. In addition, a heuristic is used to decrease the complexity of the association step of the ICP from $O(n^{2})$ to O(n). The performance of the SLAM framework is tested using two real world datasets: First, a 2.5D bathymetric dataset obtained with the usual down-looking multibeam sonar configuration, and second, a full 3D underwater dataset acquired with a multibeam sonar mounted on a pan and tilt unit.

## 1. Introduction

For Autonomous Underwater Vehicles (AUVs), addressing the navigation and mapping problems is crucial to achieve a fully operational status. Dead reckoning navigation systems suffer from an unbounded drift error, regardless of using high-end Internal Navigation Systems (INS) [1]. To avoid this, such systems are commonly aided with absolute positioning fixes. Using the measurements from a Global Positioning System (GPS) receiver is a typical solution during operations taking place on the surface. When the vehicle is submerged, Long Base Line (LBL) systems [2] can be used for the same purpose, although complex calibration of the acoustic beacon network is required prior to its operation. Using single beacon/transponder methods may reduce the calibration burden [3,4] or even eliminate it, at the cost of a reduced accuracy, when inverted LBL [5] or Ultra Short Base Line (USBL) [6] systems are used instead.

All those methods share the limitation of confining the robot operation to the area of coverage of the system. Terrain-based navigation (TBN) methods [7] can mitigate this limitation when an *a priori* Digital Terrain Map (DTM) is available on the target area. However, for an underwater vehicle to become truly autonomous, it should be able to localize itself using only on-board sensors and without the help of any external infrastructure. The Simultaneous Localization And Mapping (SLAM) concept aims to achieve that. Although more than 20 years of research have provided different approaches to solve the SLAM problem, mostly in land mobile robotics [8], there are still few solutions for underwater use, mainly due to the sensing limitations imposed by the medium and the complexity of the environment.

Underwater SLAM can be divided in two main categories: sonar and vision based SLAM. Although vision sensors may suffer from poor visibility in turbid waters, they provide fast refresh rates and high-resolution data at a fraction of the cost of a sonar sensor. Several noteworthy examples of underwater visual SLAM have been presented during recent years [9,10,11,12]. On the other hand, sonar sensors can work in bad visibility conditions, penetrating further (10–150 m) because of the low attenuation of sound in water. However, the refresh rate and resolution are medium to low and are generally expensive. Although the number of underwater SLAM examples using sonar is still reduced, they are promising.

Regarding imaging sonar mosaicking, [13] presented a feature-based registration method for two-dimensional forward-looking sonar images, while [14] developed a Fourier-based registration method to build large-scale mosaics. Moreover, several feature-based methods have been reported using: (1) point features extracted from mechanically/electronically scanned imaging sonars [15,16,17] or using a synthetic aperture imaging sonar [18]; and (2) line features extracted from a Mechanical Scanning Imaging Sonar (MSIS) in a man-made environment [19]. However, it is extremely difficult to extract features robustly in a natural underwater environment. Therefore, some researchers have focused on using featureless methods such as scan matching or occupancy grids. The work presented in [20] proposed a SLAM algorithm using a Particle Filter (PF) and range measurements from multiple pencil-beam sonars to generate an occupancy grid of a sinkhole. The method was time and computationally efficient because of the use of an octree structure to represent the environment. Although bathymetric (elevation 2.5D) maps are commonly used in the context of TBN, there have been few studies reporting successful SLAM implementations using bathymetric maps generated with data from a multibeam profiler. The pioneering work in [21] used cross correlation and Iterative Closest Point (ICP) for coarse and fine registration of bathymetric surfaces. More recently, [22] presented the bathymetric distributed particle SLAM (BPSLAM), an algorithm based on the distributed particle SLAM (DPSLAM) [23], which used a PF similar to the one proposed in [20] but representing the environment as a bathymetric map distributed across the ancestry of a given particle. It is worth mentioning that those methods were specifically designed for 2.5D elevation data, and, therefore, they are not suited for full 3D underwater environments.

This paper presents the extension to 3D of the work previously presented in [24]. The registration algorithm is a 3D-capable evolution of the 2D MSIS probabilistic Iterative Correspondence (MSISpIC) algorithm [25], which has been already applied to 2D SLAM in underwater man-made [26,27] and natural [28] environments. Our method is similar to the previous work of [21] but takes advantage of recent results obtained using the probabilistic ICP algorithms mentioned above, which are better suited to dealing with the uncertainty inherent in sonar data. Moreover, our method is not restricted to using solely 2.5D bathymetric data, and, hence, new results obtained with full 3D data are also reported here.

The 3D underwater SLAM framework presented here corrects the robot trajectory in order to produce high consistency underwater maps. The algorithm, like other state of the art SLAM techniques [21], divides the mission into a set of submaps, or surface patches, created by combining multibeam data and an estimate of the navigation until certain criteria are fulfilled. Every time a submap is created, possible overlaps with other existing patches are checked to look for loop closures. If any are found, the registration process takes place between the patches in order to refine the robot navigation. One of the novelties of the proposed method is the implementation of a two-step Probabilistic ICP (pICP) with point-to-point and point-to-plane for rough and fine registration, respectively. The improved registration method also incorporates a point cloud subsampling strategy to decrease the number of involved points as well as a novel method to decrease the complexity in the association step of the pICP from $O(n^{2})$ to O(n).

The rest of the paper is organized as follows. First, Section 2 focuses on the submap creation. In Section 3, the registration algorithm is explained, followed by the SLAM algorithm in Section 4. Section 5 presents the experiments and results, and, finally, Section 6 presents the conclusions and future work.

## 2. Submap Creation

**Figure 1:**
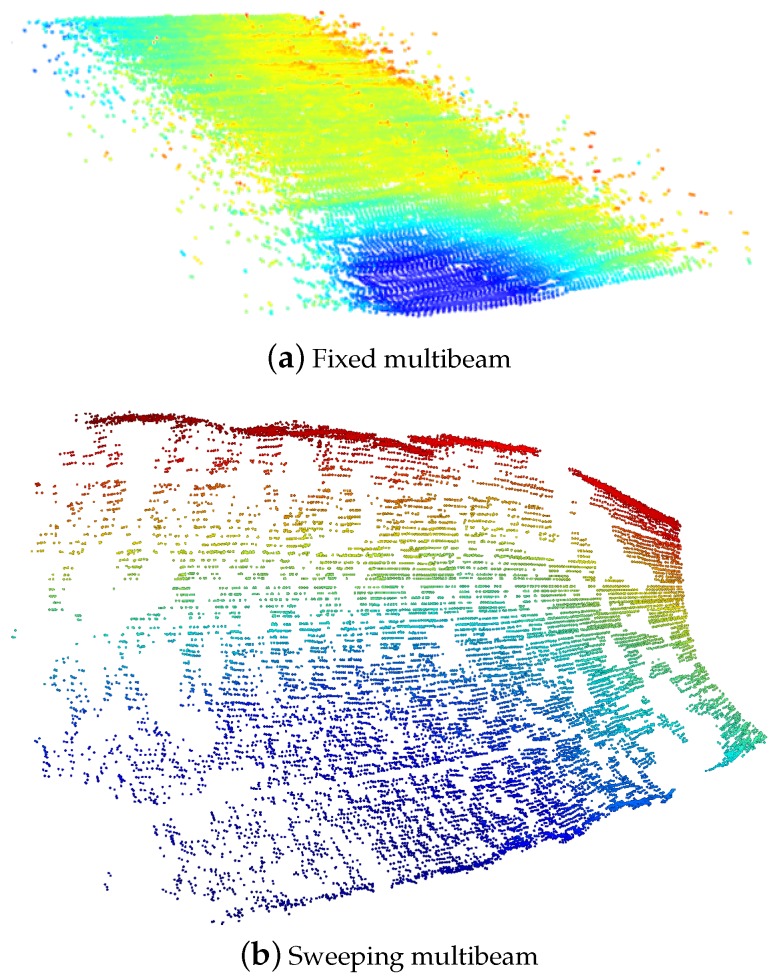
Two different submaps colored according to depth. In (**a**), the multibeam was mounted in a fixed downward-looking configuration, typically from bathymetric mapping; In (**b**), the sonar head was mounted on a pan and tilt unit and swept vertically to cover a portion of steep terrain.

For bathymetric mapping, multibeam sonars are generally fixed to the vehicle so that the 2D swath profiles are generated perpendicular to the surge direction. In that way, 2.5D surfaces are built by composing the multibeam data with the displacement of the vehicle. Alternatively, more complex environments can be inspected by sweeping a multibeam sonar mounted on a pan and tilt unit, so it is the rotation of the sonar head, and not solely the vehicle motion, that leads to the coverage of the surfaces. The point clouds resulting from the collection of multibeam data (Figure 1), along with other information such as boundaries or position with respect to the world, are what we refer to in this work as patches or submaps. This section describes the process of building these submaps during a mission.

### 2.1. Dead Reckoning

To be able to construct the submaps, regardless of whether the sonar is being swept or mounted on a fixed position, it is necessary to estimate the AUV position at the time each multibeam reading was acquired. As will be later detailed in Section 2.2, our procedure uses the relative displacements made by the vehicle between consecutive multibeam swaths to compound the point clouds. Moreover, given the probabilistic nature of the proposed registration algorithm, it is also necessary to estimate how the uncertainty evolves during these motions. To obtain this information, an Extended Kalman Filter (EKF) is used.

The state vector of the filter (see Equations (1) and (2)) contains 12 elements representing the current six Degrees of Freedom (DoF) vehicle position and velocity, as well as two more elements corresponding with the stored *x* and *y* position of the vehicle at the time when the last multibeam reading was obtained ($x_{mb},y_{mb}$):

(1) $${\hat{x}}_{k}={\left[\begin{array}{cccccccccccccc} x & y & z & \phi & \theta & \psi & u & v & w & p & q & r & x_{mb} & y_{mb} \end{array}\right]}_{k}^{T}$$

(2) $$P_{x_{k}}=E{\left(\left[x_{k}-{\hat{x}}_{k}\right]{\left[x_{k}-{\hat{x}}_{k}\right]}^{T}\right)}^{T}$$

A constant velocity kinematic model is used for prediction of the vehicle states, while those regarding the stored previous vehicle position are assumed static. In the correction stage, updates are performed asynchronously with the measurements coming from an Attitude and Heading Reference System (AHRS), a Doppler Velocity Log (DVL), and a pressure sensor. The filter iterates normally until a new multibeam reading is received. When this occurs, one last prediction is made to get an updated estimation of the vehicle’s position before calculating $o_{k}=N\left({\hat{o}}_{k},P_{o_{k}}\right)$, a new vector containing the displacement executed by the vehicle in the horizontal plane during the period of time between the current and the previous multibeam readings, as well as the *z* position and orientation of the vehicle at the current time:

(3) $${\hat{o}}_{k}={\left[\begin{array}{cccccc} x-x_{mb}, & y-y_{mb}, & z, & \phi , & \theta , & \psi \end{array}\right]}_{k}^{T}$$

(4) $$P_{o_{k}}=J_{o}P_{x_{k}}J_{o}^{T};\;\;J_{o}=\left[\begin{array}{cccc} I_{2\times 2} & 0_{2\times 4} & 0_{2\times 6} & -I_{2\times 2}\\ 0_{4\times 2} & I_{4\times 4} & 0_{4\times 6} & 0_{4\times 2} \end{array}\right]$$

Note that the two first elements of $o_{k}$ correspond to incremental values, while the other four are absolute with respect to the base reference frame used for the dead reckoning. The calculation of those increments is motivated by the cumulative drift that affects the motion in the horizontal plane. Since those states are only estimated indirectly by the velocity measurements from the DVL, the uncertainty in the xy position grows without bound. As will be introduced in the following section, working with those increments allows for a better distribution of the uncertainties within the point cloud. On the other hand, the remaining states in $o_{k}$ are observed directly by other sensors (the *z* position is observed by the pressure sensor, and the orientation by the AHRS), and therefore their uncertainties are bounded.

Once $o_{k}$ has been calculated, it is stored until the current submap is finalized. To continue with the execution of the dead reckoning filter, and to keep track of the displacements from the current position to that of the next multibeam measurement, it is necessary to replace the last two elements of the state vector ($x_{mb},y_{mb}$) with the current position of the vehicle *x* and *y*:

(5) $${\hat{x}}_{k}^{*}={\left[\begin{array}{cccccccccccccc} x & y & z & \phi & \theta & \psi & u & v & w & p & q & r & x & y \end{array}\right]}_{k}^{T}$$

(6) $$P_{x_{k}^{*}}=J_{o}^{*}P_{x_{k}}J_{o}^{*T};\;\;J_{o}^{*}=\left[\begin{array}{cc} I_{12\times 12} & 0_{12\times 2}\\ I_{2\times 2} & 0_{2\times 12} \end{array}\right]$$

Given that, the execution of the filter can continue by replicating the procedure we have just described.

### 2.2. Submap Forming

During the execution of the mission, the information required for the generation of the patches is stored in a temporal data structure $S_{temp}$:

(7) $$S_{temp}=\left\{\begin{array}{ccc} O & M & R \end{array}\right\}$$

where $O=\{o_{1},...,o_{n}|o_{i}=N\left({\hat{o}}_{i},P_{o_{i}}\right)\}$ is the set of displacements and positions as computed in Equations (3) and (4), while $M=\{m_{1},...,m_{n}\}$, with $m_{i}=\left\{\delta_{1},...,\delta_{m}|\delta_{i}=N\left({\hat{\delta }}_{i},P_{\delta_{i}}\right)\right\}$, is the set of all the multibeam swaths ***m***, each one containing the corresponding polar range measurements δ. Finally, $R=\{r_{1},...,r_{n}|r_{i}=N\left({\hat{r}}_{i},P_{r_{i}}\right)\}$ is the set of transformations required to represent the multibeam data with respect to the vehicle frame. This is particularly relevant in the case of a multibeam sonar mounted on a pan and tilt unit, since the transformations will change continuously because of the sweeping motion.

When the amount of accumulated data is deemed sufficient (see the conditions below), the current patch is closed and the contents of $S_{temp}$ are used to generate the point cloud and other information that will be necessary later during the registration process. In addition, the position of the recently terminated patch is stored in the state vector of the pose-based EKF in charge of the SLAM process (see Section 4). Before beginning a new patch, the $S_{temp}$ is reset to store a new batch of data.

The criteria to close a patch depend on which scenario we are dealing with. If the sonar is scanning a tri-dimensionally rich environment by means of a pan and tilt unit, each complete sweep is taken as an independent submap because, unless a very fast vehicle is used, successive scans will re-visit the same area, which only contributes to increasing the number of points without incorporating significant new information. On the other hand, the situation with typical bathymetric survey missions where the multibeam is fixed on the vehicle is substantially different. Scanned areas are generally not re-visited (not in the same transect), and the seabed is often scarce in features, which may make the successful matching of surface patches difficult. In this case, a combination of three criteria is used to determine when to close a patch:

- **Minimum size:** A minimum size is defined to avoid handling a large number of tiny patches augmenting unnecessarily the length of the SLAM state vector and reducing the overlapping.
- **Maximum size:** The maximum size is bounded to avoid handling huge patches with a high uncertainty in the surface points due to the accumulated dead reckoning error.
- **Normal occupancy:** The surface relief is analyzed to determine when the patch is rich enough to be successfully matched. The procedure basically consists in finding surface normals for each point on the cloud and representing their parametrization on a histogram. If the histogram is sufficiently occupied, the submap is closed.

**Figure 2:**
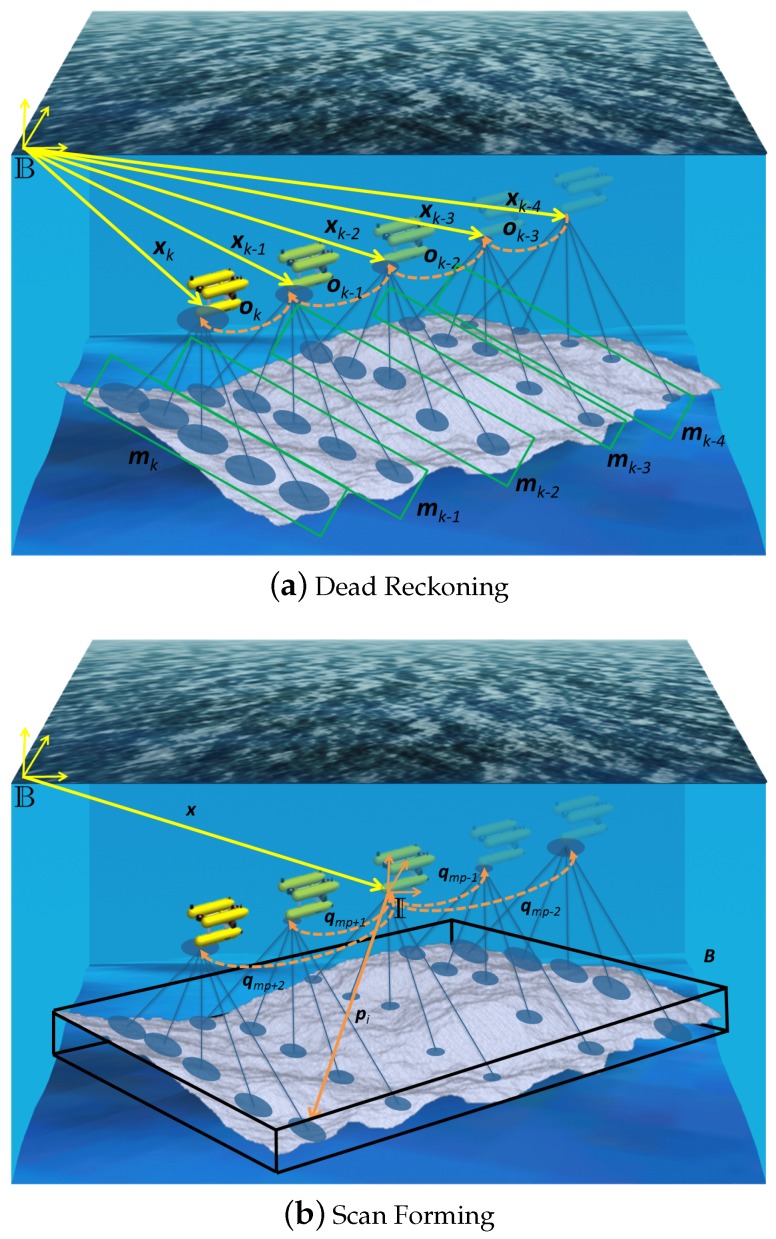
Example of the dead reckoning (**a**) and the scan forming (**b**).

Once sufficient data has been acquired and the submap is closed, all the stored data is processed to generate the point cloud and the information required for the potential registration with other submaps. In [21], the reference frame for each submap was defined as the position of the robot when the patch was started. Here, the point cloud is generated with respect to a new reference frame I, which is placed on top of the central position of the trajectory executed during the creation of the patch, but oriented like the base frame B used for the dead reckoning navigation. By placing this frame in the center of the submap, the uncertainty of the points grows from the center of the patch to the edges (see Figure 2b) instead of growing from the beginning to the end (see Figure 2a). This gives a more convenient distribution of the uncertainty in the point cloud which improves the registration [29].

The process of generating the point cloud begins by selecting the central position that will be associated to I, and that will be referenced hereafter with the mp subindex. Then, the $q_{k}$ vector relating a given *k* position in which a multibeam reading was acquired, and the I reference frame can be computed from the corresponding $o_{k}$ and $o_{mp}$ (both pertaining to ***O*** and stored in $S_{temp}$) as:

(8) $${\hat{q}}_{k}=\left\{\begin{array}{ccc} {\left[\begin{array}{cccccc} x_{q_{k-1}}+x_{o_{k}}, & y_{q_{k-1}}+y_{o_{k}}, & z_{o_{k}}-z_{o_{mp}}, & \phi_{o_{k}}, & \theta_{o_{k}}, & \psi_{o_{k}} \end{array}\right]}^{T} & & k>mp\\ {\left[\begin{array}{cccccc} 0, & 0, & 0, & \phi_{o_{k}}, & \theta_{o_{k}}, & \psi_{o_{k}} \end{array}\right]}^{T} & & k=mp\\ {\left[\begin{array}{cccccc} x_{q_{k+1}}-x_{o_{k+1}}, & y_{q_{k+1}}-y_{o_{k+1}}, & z_{o_{k}}-z_{o_{mp}}, & \phi_{o_{k}}, & \theta_{o_{k}}, & \psi_{o_{k}} \end{array}\right]}^{T} & & k<mp \end{array}\right.$$

where $x_{a}$ should be read here as the element *x* contained in the vector ***a***. Note that for computing $q_{k}$, the vectors $q_{k-1}$ (if k>mp) or $q_{k+1}$ (if k<mp) also need to be known. This means that the calculation needs to be done sequentially starting by the mp position and then moving towards both ends of the submap (1 and *n*). The uncertainty of $q_{k}$ is then computed as:

(9) $$P_{q_{k}}=\left\{\begin{array}{ccc} J_{1}P_{q_{k-1}}J_{1}^{T}+J_{2}P_{o_{k}}J_{2}^{T}+J_{3}P_{o_{mp}}J_{3}^{T} & & k>mp\\ J_{4}P_{o_{k}}J_{4}^{T} & & k=mp\\ J_{1}P_{q_{k+1}}J_{1}^{T}+J_{5}P_{o_{k+1}}J_{5}^{T}+J_{3}P_{o_{mp}}J_{3}^{T}+J_{4}P_{o_{k}}J_{4}^{T} & & k<mp \end{array}\right.$$

being $J_{j}$ the Jacobians of the function:

(10) $$\begin{array}{cc} J_{1}= & \left[\begin{array}{cc} I_{2\times 2} & 0_{2\times 4}\\ 0_{4\times 2} & 0_{4\times 4} \end{array}\right] \end{array}$$

(11) $$\begin{array}{cc} J_{2}= & I_{6\times 6} \end{array}$$

(12) $$\begin{array}{cc} J_{3}= & \left[\begin{array}{ccc} 0_{2\times 2} & 0_{2\times 1} & 0_{2\times 3}\\ 0_{1\times 2} & -1 & 0_{1\times 3}\\ 0_{3\times 2} & 0_{3\times 1} & 0_{3\times 3} \end{array}\right] \end{array}$$

(13) $$\begin{array}{cc} J_{4}= & \left[\begin{array}{cc} 0_{3\times 3} & 0_{3\times 3}\\ 0_{3\times 3} & I_{3\times 3} \end{array}\right] \end{array}$$

(14) $$\begin{array}{cc} J_{5}= & -J_{1} \end{array}$$

It is worth noting that $q_{k}$ is composed of both relative (first three elements representing a displacement) and absolute (last three elements being the orientation) measurements. Assuming that correlations in attitude estimates are negligible, computing the relative increment of the orientation would end up adding uncertainty in these 3 DoF artificially. This would apply also to the *z* displacement. However, we observed that if the same approach is taken in this DoF, the lever-arm effect in the registration process (the depth is referenced to the water surface) makes it much more prone to error. Therefore, we decided to reference the *z* position to the actual depth of the AUV regardless of the increment of uncertainty in order to make the registration process more stable.

With all the $q_{k}$ computed, the point cloud can now be generated. The first step is to transform all the polar range measurements $\delta_{i}=N\left(\hat{\delta_{i}},P_{\delta_{i}}\right)$ which are represented in the sensor frame to that of the vehicle using the $r_{k}$ transformations stored in ***R***:

(15) $${\hat{p}}_{i}^{\times }={\hat{r}}_{k}⊕g\left({\hat{\delta }}_{i}\right)$$

(16) $$P_{p_{i}^{\times }}=J_{1⊕}\;P_{r_{k}}J_{1⊕}^{T}+J_{2⊕}\left(J_{g}P_{\delta }J_{g}^{T}\right)J_{2⊕}^{T}$$

where g(.) is the polar to Cartesian conversion function, $J_{g}$ is its corresponding Jacobian and ⊕ is the compounding operator with Jacobians $J_{1⊕}$ and $J_{2⊕}$ as defined in [30]. With the point $p_{i}^{\times }$ referenced to the vehicle frame and $q_{k}$ being the vehicle position referenced to I, we can calculate the position of a point $p_{i}$ as:

(17) $${\hat{p}}_{i}={\hat{q}}_{k}⊕{\hat{p}}_{i}^{\times }$$

(18) $$P_{p_{i}}=J_{1⊕}\;P_{q_{k}}J_{1⊕}^{T}+J_{2⊕}P_{p_{i}^{\times }}J_{2⊕}^{T}$$

After all these calculations, the information regarding the patch is saved in a new structure ***S*** as:

(19) $$S=\left[\begin{array}{cccc} x & O & W & B \end{array}\right]$$

where $x=N(\hat{x},P_{x})$ is the position of the frame I that will be used to anchor the submap in the state vector of the pose-based SLAM framework described in Section 4; ***O*** is the same set of transformations as in $S_{temp}$; $W=\{p_{1},...,p_{n}|\;p_{i}=N\left({\hat{p}}_{i},P_{p_{i}}\right)\}$ is the set of points referenced to I that have been calculated and, finally, ***B*** is a volume containing all the points that pertain to the patch. On the horizontal plane, ***B*** is the polygon containing all the ${\hat{p}}_{i}$ points, while, on the *z* direction, the boundary is defined by the minimum and maximum depth of all the points ${\hat{p}}_{i}$ (see Figure 2).

## 3. Registration Algorithm

**Figure 3:**
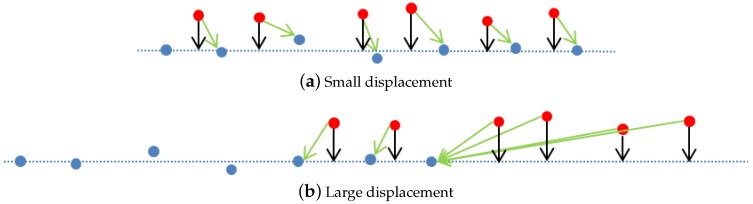
Point-to-point and point-to-plane comparison in the presence of small displacement (**a**) and large displacement (**b**). In blue, the points of the reference scan with the plane, in red, the points of the new scan. Green arrows correspond to point-to-point association while black ones represent point-to-plane.

This section explains the procedure to register two submaps using probabilistic ICP. The inputs of the algorithm are a reference submap $S_{ref}$, which has been already stored in the SLAM framework; a newly generated submap $S_{new}$, and an initial guess of their relative displacement $q_{0}=N\left(\hat{q_{0}},P_{q_{0}}\right)$ obtained from the navigation. The algorithm uses a two-stage correction procedure. First, a point-to-point correction is performed to roughly align the two submaps (until their relative displacement in two consecutive iterations falls below a threshold), and then, a point-to-plane correction is executed to refine the result. Point-to-point association tends to produce undesired effects in the presence of small misalignments (see for instance the lateral displacement depicted in Figure 3a). This is because associated points do not necessarily correspond to the exact same spot in the original surface and therefore their arbitrary occurrence may prevail over the general shape of the surface. However, point-to-point association is powerful when large displacements are present (see Figure 3b). On the other hand, point-to-plane associations tend to be driven by the shape of the surface and hence, perform better in the presence of small misalignments (see Figure 3a), but may fail when dealing with large displacements (see Figure 3b). To complement their strengths and weaknesses, we combine both methods by using an error threshold which determines when to switch from one strategy to the other.

### 3.1. Point-to-Point Association

Given a certain point $n_{i}=N\left({\hat{n}}_{i},P_{n_{i}}\right)$ from the new patch $S_{new}$, and a matching candidate $r_{j}=N\left({\hat{r}}_{j},P_{r_{j}}\right)$ from the reference surface $S_{ref}$, both represented in Cartesian coordinates and referenced to their respective frames ($I_{new}$ and $I_{ref}$), the association error $e_{ij}=N\left({\hat{e}}_{ij},P_{e_{ij}}\right)$ can be defined as:

(20) $${\hat{e}}_{ij}=\hat{r_{j}}-\hat{q_{0}}⊕{\hat{n}}_{i}$$

(21) $$P_{ij}=P_{r_{j}}+J_{1⊕}P_{q_{0}}J_{1⊕}^{T}+J_{2⊕}P_{n_{i}}J_{2⊕}^{T}$$

so the point-to-point association may be solved through a simple individual compatibility test over the corresponding Mahalanobis distance:

(22) $$\begin{array}{c} d^{2}={\hat{e}}_{ij}^{T}P_{ij}^{-1}{\hat{e}}_{ij}<\chi_{d,\alpha }^{2} \end{array}$$

All the points individually compatible with $n_{i}$ form the set $A_{i}$. From this set, the one with smaller Mahalanobis distance is chosen to be associated with $n_{i}$.

### 3.2. Point-to-Plane Association

At the second stage, the metric changes and the point-to-plane distance is used instead. Now, the set of compatible points $A_{i}$ is used to estimate a local plane $\Pi (\nu_{i},d_{i})$ whose equations are given by $\nu_{i}^{T}x-d_{i}=0$, being $d_{i}$ the plane distance to the origin and $\nu_{i}$ its normal vector. Because of the probabilistic nature of our algorithm, we are interested not only in the plane parameters but also in their uncertainty. An iterative method is reported in [31] for this purpose, being too computationally expensive for our case. In [32], the authors use a two-step minimization method for estimating: (1) the plane using region growing algorithms and (2) its uncertainty. Finally, in [33], the error of a set of samples is minimized using the uncertainty related to the range of the sensor by means of a weighted Principal Component Analysis (PCA). This last method is the one which best fits our requirements because of its reduced computational complexity, and also because of its nature, since it does not search for the points forming the plane, but fits the plane among the given points.

Given a plane Π(ν,d), whose equation is $\nu^{T}x=d$, the likelihood of observing a plane point $r_{j}\in A_{i}$ is given by:

(23) $$p\left(r_{j}|\nu ,d\right)=\frac{1}{\sqrt{2\pi }\left|P_{r_{j}}\right|}\exp\left(\left(\nu^{T}r_{j}-d\right)*\nu {)}^{T}P_{r_{j}}^{-1}(\left(\nu r_{j}-d\right)*\nu \right)$$

The objective here is to maximize the sum of the log-likelihood of the previous equation. The problem cannot be solved in a simple way since the error of the uncertainty depends twice on the normal ν. To solve the problem in an efficient way, it was necessary to approximate the uncertainty by the trace of $P_{r_{j}}$: $\mathrm{Tr}\left(P_{r_{j}}\right)$. In this way, the error ellipsoid is approximated to a sphere, and it is possible to solve the equation analytically and as efficiently as in [33] (please refer to this work for a more extended derivation).

The log-likelihood that we want to maximize, ignoring constants, is the approximate least squares problem:

(24) $$\ell =\underset{\nu ,d}{\mathrm{argmax}}\;-\frac{1}{2}\sum_{i=1}^{N}\frac{{\left(\nu^{T}r_{j}-d\right)}^{2}}{\mathrm{Tr}{\left(P_{r_{j}}\right)}^{2}}$$

with Lagrangian

(25) $$L=-\frac{1}{2}\sum_{i=1}^{N}\frac{{\left(\nu^{T}r_{j}-d\right)}^{2}}{\mathrm{Tr}{\left(P_{r_{j}}\right)}^{2}}-\lambda \left(\nu^{T}\nu -1\right)$$

Setting $\frac{\partial L}{\partial d}=0$, we find the solution

(26) $$d_{⋆}=\nu_{⋆}^{T}p_{\mu },\;\mathrm{with}\;\;p_{\mu }=\left(\sum_{i=1}^{N}\mathrm{Tr}{\left(P_{r_{j}}\right)}^{-2}r_{j}\right){\left(\sum_{i=1}^{N}\mathrm{Tr}{\left(P_{r_{j}}\right)}^{-2}\right)}^{-1}$$

$p_{\mu }$ being the weighted center of the set of points $A_{i}$. Finally,

(27) $$\nu_{⋆}=\underset{\nu }{\mathrm{argmin}}\;\nu^{T}\left(\sum_{i=1}^{N}\frac{\left(r_{j}-p_{\mu }\right){\left(r_{j}-p_{\mu }\right)}^{T}}{\mathrm{Tr}{\left(P_{r_{j}}\right)}^{2}}\right)\nu$$

The minimizing normal $\nu_{⋆}$ is defined by the eigenvalues of the covariance matrix of the points as in the common weighted PCA method. The uncertainty of the estimator is found as:

(28) $$P_{f}=-H^{+}=\left(\begin{array}{cc} P_{\nu } & P_{\nu }P_{d}\\ P_{d}P_{\nu } & P_{d}, \end{array}\right)$$

where ***H*** is the Hessian of the Lagrangian in the optimal plane.

Given the point $n_{i}$ and the plane $\Pi_{i}\left(\nu_{i},d_{i}\right)$ estimated from all the compatible points in $A_{i}$, the point $a_{i}$ is defined as the orthogonal projection of $n_{i}$ over the plane $\Pi_{i}\left(\nu_{i},d_{i}\right)$:

(29) $$\hat{a_{i}}={\hat{q}}_{0}⊕{\hat{n}}_{i}-\left({\left({\hat{q}}_{0}⊕{\hat{n}}_{i}\right)}^{T}{\hat{\nu }}_{i}-{\hat{d}}_{i}\right){\hat{\nu }}_{i}$$

(30) $$P_{a_{i}}=\frac{\partial a_{i}}{\partial q_{0}}P_{q_{0}}{\frac{\partial a_{i}}{\partial q_{0}}}^{T}+\frac{\partial a_{i}}{\partial n_{i}}P_{n_{i}}{\frac{\partial a_{i}}{\partial n_{i}}}^{T}+\frac{\partial a_{i}}{\partial \nu_{i}}P_{\nu_{i}}{\frac{\partial a_{i}}{\partial \nu_{i}}}^{T}+\frac{\partial a_{i}}{\partial d_{i}}P_{d_{i}}{\frac{\partial a_{i}}{\partial d_{i}}}^{T}$$

This new virtual point $a_{i}$ is actually the point that will be associated with $n_{i}$ to execute the new registration phase using the same point-to-point equations we already presented in Equations (20) and (21), but using $a_{i}$ instead of $r_{j}$.

### 3.3. Minimization

At the end of each association stage, a minimization process is executed to estimate the robot displacement $q_{\min}$ that minimizes the addition of the Mahalanobis distance of the association error:

(31) $$\begin{array}{c} q_{\min}=\underset{q}{\mathrm{argmin}}\sum \left\{\xi P_{\xi }^{-1}\xi \right\} \end{array}$$

ξ being a vector composed of all the ${\hat{e}}_{ij}$ error vectors (see Equation (20)) after associating all the points (either virtual or real) and $P_{\xi }$ the block diagonal matrix with their corresponding covariances $P_{e_{ij}}$ (Equation (21)). This minimization is done using weighted least squares:

(32) $$\begin{array}{c} q_{\min}={\left[J^{T}P_{\xi }^{-1}J\right]}^{-1}J^{T}P_{\xi }^{-1}\xi \end{array}$$

***J*** being the Jacobian matrix of the error function at the previous estimation evaluated in all the points.

### 3.4. Submap Simplification

**Figure 4:**
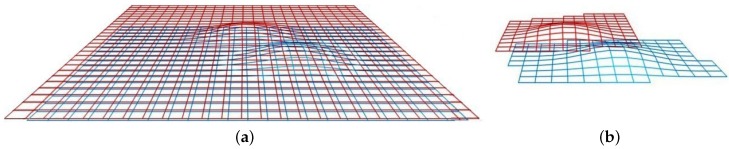
A 3D visual concept of the idea behind octree use: two surfaces to be matched (**a**); the same surfaces resampled (**b**).

Traditional ICP-based methods may encounter some problems in a scenario like the one depicted in Figure 4a, where two almost flat surfaces share a poorly visible feature. For instance, ICP tends to associate each point with its closest neighbour according to a particular metric. Because of that, it may be difficult to correctly associate the feature areas when the displacement is large (*i.e.*, they are far from each other, and the proximity of flat areas may lead to a local minimum). This particular issue will benefit from the proposed probabilistic ICP approach, since the uncertainty of the points should constrain the possible matching candidates to those compatible with the real accumulated error. For instance, uncertainties may be large in the horizontal plane, making it possible to match two distant features, but small in the z direction, so points in the flat areas will not be compatible with those in the features.

Another inconvenience related to traditional ICP-based methods is the weak contribution of flat areas to the registration (they all look alike and their matching possibilities are high). Moreover, when few features are present in scenarios of large flat areas, the planar areas may prevail and lead to poor matching. ICP algorithms have better results when the associated points are significant (*i.e.*, distinguishable from each other). For that reason, a new sampling procedure to reduce the number of points in the cloud by removing the less informative ones is presented (see Figure 4b). Since the surface distribution is not available, the sampling procedure is performed using the discrete points. This resampling improves the odds of successful matchings, even when large displacements are present, as well as decreasing the computation time in the registration by drastically decreasing the number of points to be associated, thus increasing the performance of the algorithm.

**Figure 5:**
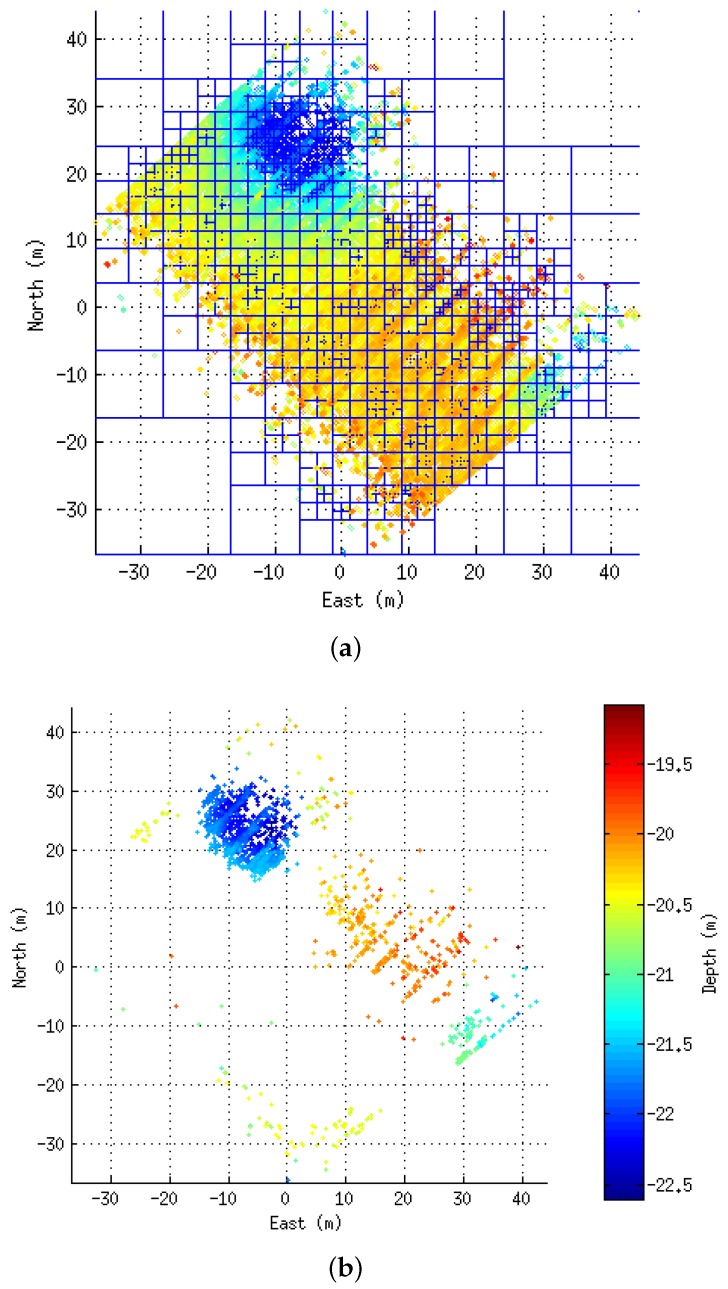
The contribution of octree in point resampling: octree construction, top view (**a**); points after resampling (**b**).

The approach proposed here uses an octree structure to sample the scan in its most significant areas (*i.e.*, areas with rich relief). The subsampling algorithm works as follows: the point cloud is contained in a discretized tridimensional space structured as an octree. Using the points contained in each cell of the octree, a relief-based subsampling criteria is evaluated recursively, and if the condition is fulfilled, the cell is divided into eight subcells. After the subdivision process comes to an end, only one point is taken from each cell of the octree (see Figure 5). This makes areas with bigger (*i.e.*, not significant) cells contribute with fewer points than areas with smaller (*i.e.*, significant) cells. In [34], several different criteria were studied to drive the octree subdivision. Although some criteria were more suitable for specific types of environments, in this work, the *difference from principal plane* method has been selected given its overall performance in both 2.5D and 3D. This criteria basically dictates that a cell should be divided if the average distance between the points in the cell, and the best fitting plane of the cloud is higher than a given threshold. For a more detailed description, please refer to [34].

### 3.5. Association in Linear Time

The association step in ICP based methods has an $O(n^{2})$ computational cost because it is necessary to compare each reference point $r_{j}$ in $S_{ref}$ against all the $n_{i}$ points in $S_{new}$ to compute their distances. Moreover, the probabilistic implementation of the ICP method requires several matrix operations, including an inversion, to calculate the uncertainty of the association of the points from the two point clouds. Hereafter, a new method for reducing complexity taking advantage of the uncertainty estimates of the points, which are already available, is proposed.

A probabilistic point ***p*** with uncertainty ***P*** can be represented graphically as an ellipsoid defined by a $\chi^{2}$ distribution at a certain confidence level *α* and for *d* degrees of freedom (DoF):

(33) $$\left\{(x|{\left(x-p\right)}^{T}P^{-1}\left(x-p\right)=\chi_{d,\alpha }^{2}\right\}$$

**Figure 6:**
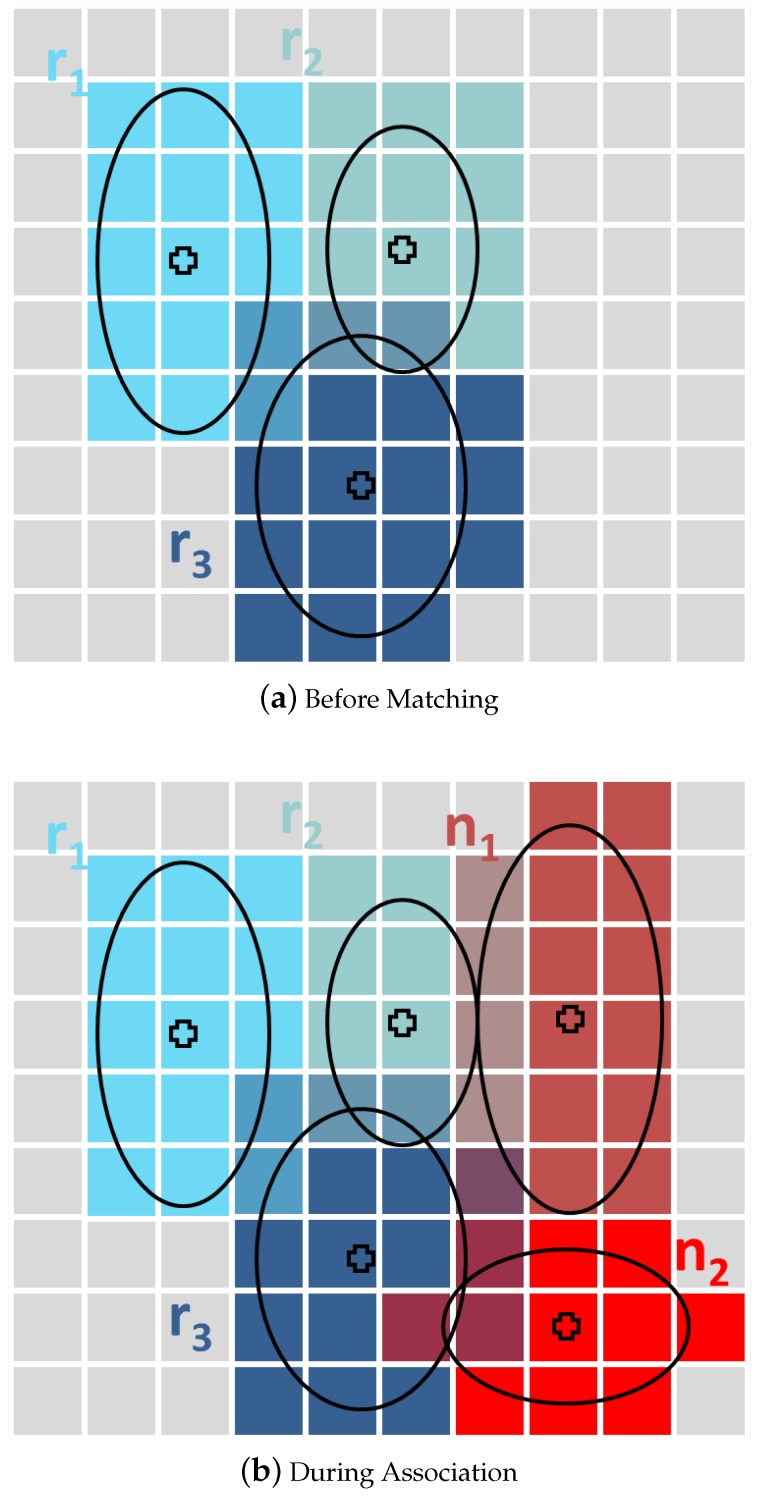
The figure shows how the support grid is used during the association step. First, the points in the reference scan (**blue**) are inserted into the cells using their uncertainty ellipses (**a**). Then, each point in the new scan **(red**) is also laid inside the grid (**b**). In this case, $n_{1}$ overlaps with $r_{2}$ and $r_{3}$ while $n_{2}$ overlaps only with $r_{3}$. Moreover, $r_{1}$ has no potential associations.

Given that, in our approach, a 3D grid is generated covering the two patches to be matched, and for each point in $S_{ref}$, the cells falling inside its uncertainty ellipse are marked (see Figure 6a). During the association process, the same procedure is followed for each point in $S_{new}$.

At this point, the following heuristic is applied: To check the compatibility of two points $n_{i}$ and $r_{j}$, we define their ellipsoids given a certain confidence level *α*. If the ellipsoids do not intersect with each other, the corresponding points are assumed not to be individually compatible. In other words, if:

(34) $$\begin{array}{c} \left\{(x|{\left(x-n_{i}\right)}^{T}P_{n_{i}}^{-1}\left(x-n_{i}\right)=\chi_{d,\alpha }^{2}\right\}\cap \\ \left\{(x|{\left(x-r_{j}\right)}^{T}P_{r_{j}}^{-1}\left(x-r_{j}\right)=\chi_{d,\alpha }^{2}\right\}=\emptyset \end{array}$$

then,

(35) $$\left(n_{i}-r_{j}\right){\left(P_{n_{i}}+P_{r_{j}}\right)}^{-1}{\left(n_{i}-r_{j}\right)}^{T}>\chi_{d,\alpha }^{2}$$

Note that evaluating the compatibility in this way is still computationally expensive. However, in our method, the space occupied by the ellipses has been previously registered inside a grid, so it is possible to rapidly find the intersecting ellipsoids using a direct grid look-up (see Figure 6b). In other words, association candidates for a given point in the new scan can be easily identified by searching only among the cells occupied by its own ellipse, for tags denoting occupancy of those same cells by ellipses corresponding to points in the reference scan. In that way, candidates are directly determined, without a need to evaluate all the remaining points in the reference scan, and thus, the complexity is reduced to O(n).

## 4. SLAM Algorithm

This section describes the EKF implementation of the pose-based SLAM framework in charge of optimizing the surface map.

Every time a submap is finished, the estimate of the robot pose at the reference point of each surface patch $x_{S}$ is incorporated to the state vector *x* so it contains all the information regarding the submap distribution:

(36) $${\hat{x}}_{k}={\left[{\hat{x}}_{S_{n}}\;\cdots \;{\hat{x}}_{S_{1}}\right]}_{k}^{T}$$

with a pose state $x_{S}$ being:

(37) $$x_{S}\;=\;{\left[x\;y\;z\;\phi \;\theta \;\psi \;\right]}^{T}$$

where (x,y,z) is the position of the robot and (ϕ,θ,ψ) are the roll, pitch and yaw angles. The poses are referred to the same common frame *B* that was used during the dead reckoning. The covariance matrix for this state is defined as:

(38) $$P_{k}=E\left(\left[x_{k}-{\hat{x}}_{k}\right]{\left[x_{k}-{\hat{x}}_{k}\right]}^{T}\right)$$

### 4.1. Prediction and State Augmentation

The submap poses stored in the state vector are assumed to be static during the execution of the mission. Therefore, the prediction stage of the EKF just maintains the estimated values for the state vector and its covariance. However, every time a new patch is completed, and its pose is introduced in the state vector. This is done during the prediction stage. To be able to fit the requirements of this algorithm (such as the location of the frame I), a new procedure has been developed for the prediction and state augmentation. The procedure explained hereafter uses the previously computed $o_{k}$ to find the relationship between the patch $S_{n}$ and $S_{n+1}$ by adding all the incremental displacements in the xy plane and copying the position of the other 4 DoF at the position chosen as frame I in patch $S_{n+1}$.

Let $S_{n+1}$ be the new patch to be added to the state vector and $S_{n}$ the last one already added. Then, we need to estimate the transformation ${}^{n}q_{n+1}=N{(}^{n}{\hat{q}}_{n+1},P_{{}^{n}q_{n+1}})$ relating $S_{n}$ and $S_{n+1}$. The process begins by defining two functions that will be applied to the set of stored $o_{k}$ relationships between the two patches:

(39) $$f_{1}\left(\hat{o}\right)=f_{1}\left(\left[\begin{array}{cccccc} x, & y, & z, & \phi , & \theta , & \psi \end{array}\right]\right)=\left[\begin{array}{cccccc} x, & y, & 0, & 0 & 0, & 0 \end{array}\right]$$

(40) $$f_{2}\left(\hat{o}\right)=f_{2}\left(\left[\begin{array}{cccccc} x, & y, & z, & \phi , & \theta , & \psi \end{array}\right]\right)=\left[\begin{array}{cccccc} 0, & 0, & z, & \phi , & \theta , & \psi \end{array}\right]$$

with Jacobians:

(41) $$F_{1}=\left[\begin{array}{cc} I_{2\times 2} & 0_{2\times 4}\\ 0_{4\times 2} & 0_{4\times 4} \end{array}\right],\;F_{2}=\left[\begin{array}{cc} 0_{2\times 2} & 0_{2\times 4}\\ 0_{4\times 2} & I_{4\times 4} \end{array}\right]$$

Then, taking the stored $O=\left\{o_{1},...,o_{m}\right\}\in S_{n}$, the parameter $q_{1}=N\left(\hat{q_{1}},P_{q_{1}}\right)$ representing the distance from the central position of the $S_{n}$ patch (defined with the subindex mp) and its last position can be calculated as:

(42) $${\hat{q}}_{1}=\sum_{k=mp+1}^{m}f_{1}\left({\hat{o}}_{k}\right),\;P_{q_{1}}=\sum_{k=mp+1}^{m}F_{1}P_{o_{k}}F_{1}^{T}$$

Next, using the stored $O\in S_{n+1}$, the parameter $q_{2}=N\left(\hat{q_{2}},P_{q_{2}}\right)$ representing the distance from the beginning of $S_{n+1}$ to its center plus the final orientation of the patch can be obtained as:

(43) $${\hat{q}}_{2}=f_{2}\left({\hat{o}}_{mp}\right)+\sum_{k=1}^{mp}f_{1}\left({\hat{o}}_{k}\right),\;P_{q_{2}}=F_{2}P_{o_{mp}}F_{2}^{T}+\sum_{k=1}^{mp}F_{1}P_{o_{k}}F_{1}^{T}$$

Finally, the complete transformation ${}^{n}q_{n+1}$ relating the centers of both patches is calculated as:

(44) $${}^{n}{\hat{q}}_{n+1}={\hat{q}}_{1}+{\hat{q}}_{2},\;P_{{}^{n}{\hat{q}}_{n+1}}=P_{q_{1}}+P_{q_{2}}$$

Knowing this ${}^{n}q_{n+1}$ transformation, the state of the filter can be augmented with the new position of $S_{n+1}$ by doing:

(45) $${\hat{x}}_{k}^{+}={\left[\begin{array}{ccccc} {\hat{x}}_{S_{n}}{⊙}^{n}{\hat{q}}_{n+1} & {\hat{x}}_{S_{n}} & {\hat{x}}_{S_{n-1}} & \cdots & {\hat{x}}_{S_{1}} \end{array}\right]}_{k}^{T}$$

(46) $$P_{{\hat{x}}_{k}^{+}}=J_{1⊙}P_{{\hat{x}}_{k}}J_{1⊙}^{T}+J_{2⊙}P_{{}^{n}{\hat{q}}_{n+1}}J_{2⊙}^{T}$$

Note that the ⊙ operator is introduced here to define the way in which the global coordinates of the $S_{n}$ patch are combined with the relationship between consecutive patches $S_{n}$ and $S_{n+1}$ to find the position of the patch $S_{n+1}$ in the world frame. The ⊙ operator is described as:

(47) $${\hat{x}}_{S_{n}}{⊙}^{n}{\hat{q}}_{n+1}=\left[\begin{array}{c} x_{x_{S_{n}}}+x_{{}^{n}{\hat{q}}_{n+1}}\\ y_{x_{S_{n}}}+y_{{}^{n}{\hat{q}}_{n+1}}\\ z_{{}^{n}{\hat{q}}_{n+1}}\\ \phi_{{}^{n}{\hat{q}}_{n+1}}\\ \theta_{{}^{n}{\hat{q}}_{n+1}}\\ \psi_{{}^{n}{\hat{q}}_{n+1}} \end{array}\right]$$

with Jacobians:

(48) $$J_{1⊙}=\left[\begin{array}{c} \begin{array}{cc} I_{2\times 2} & 0_{2\times (4+6(n-1))}\\ 0_{4\times 2} & 0_{4\times (4+6(n-1))} \end{array}\\ I_{6n\times 6n} \end{array}\right],\;J_{2⊙}=\left[\begin{array}{c} I_{6\times 6}\\ 0_{6n\times 6} \end{array}\right]$$

### 4.2. Matching Strategy

**Figure 7:**
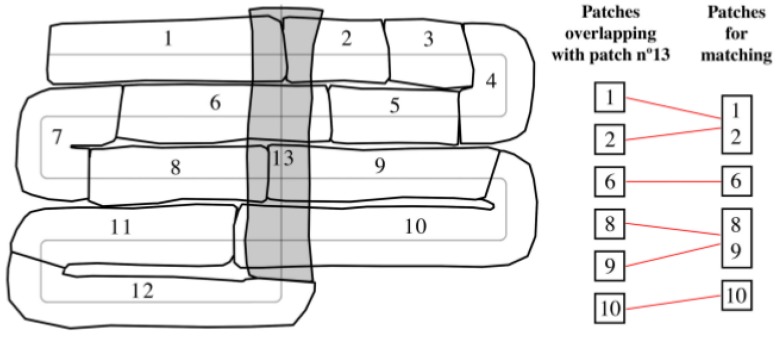
Patch number 13 overlaps with patches 1, 2, 6, 8, 9 and 10. For improving the matching process, patches that are consecutive (1 and 2 as well as 8 and 9) are merged. This results in four patches taking part in the matching process.

When a new patch is available, potential matches are searched among the previously created patches. This is done by determining the intersection between the volumes ***B*** (see Equation (19)) of the two potentially matching patches. In this way, two patches are considered to be intersecting if more than a given % of their volumes is shared. The new patch may potentially intersect with several of the patches that already exist, which may or may not be consecutive in time (see the ones overlapping with patch number 13 in Figure 7). As can also be observed, consecutive patches (such as number 1 and 2, or 8 and 9) may have a small overlap with the new patch. For this reason, a new approach is used that consists in joining consecutive patches to maximize the intersecting area. However, this is not recommended for contiguous non-consecutive patches since the drift between them might be significant (e.g., patches number 1 and 6). The proposed approach involves three steps: (1) search for patches intersecting with the new one; (2) search for consecutive patches among those previously selected; and (3) join the patches that are found to be consecutive. The resulting patches are the result of combining the points of the two surfaces and representing them in the frame I of the earliest created patch.

### 4.3. Scan Matching

In order to execute the probabilistic registration algorithm, given two overlapping scans $S_{i}$ and $S_{n}$ with related poses ${\hat{x}}_{S_{i}}$ and ${\hat{x}}_{S_{n}}$, an initial guess $q_{0}=N\left({\hat{q}}_{0},P_{q_{0}}\right)$ of their relative displacement is necessary. This can be easily extracted from the state vector using the tail-to-tail transformation:

(49) $${\hat{q}}_{0}=⊖{\hat{x}}_{S_{i}}⊕{\hat{x}}_{S_{n}}$$

where ⊕ and ⊖ are the compounding and inverse compounding operators as defined in [30]. Since the tail-to-tail transformation is actually a non-linear function of the state vector ${\hat{x}}_{k}$, the uncertainty of the initial guess can be computed using:

(50) $$P_{q_{0}}=HP_{x_{k}}H^{T}$$

where ***H*** is the Jacobian computed as:

(51) $$H=[J_{2{⊕}_{6\times 6}}\;\;0_{6\times 6(n-i-1)}\;\;J_{1⊕}J_{{⊖}_{6\times 6}}\;\;0_{6\times 6(i-1)}]$$

where $J_{1⊕}$, $J_{2⊕}$ and $J_{⊖}$ are the Jacobians of the compounding and inverse compounding functions as defined in [30]. Finally, $0_{6\times 6(n-i-1)}$ and $0_{6\times 6(i-1)}$ are zero matrices whose dimensions are determined according to the position in the state vector of the surfaces to be registered.

Once the initial displacement guess is available, the registration algorithm presented in Section 3 can be used to produce an updated measurement of this displacement.

### 4.4. State Update

The initial guess in Equation (49) defines the relationship between two patch poses in the state vector. This can be expressed by means of the following measurement model:

(52) $$z_{k}=h\left(x_{k},v_{k}\right)=⊖x_{S_{i}}⊕x_{S_{n}}+v_{k}$$

$z_{k}$ being the estimated displacement $q_{\min}$ and $v_{k}$ a zero-mean white Gaussian noise with covariance $P_{q_{\min}}$ accounting for the errors in the registration process. Given that, the standard EKF equations can be used to update the state vector.

## 5. Experiments and Results

The algorithm has been used to produce the maps for two different underwater datasets. The first one is a bathymetric (2.5D) survey carried out by the *Sirius* AUV [35] on a site of geological interest off the coast of Tasmania (Australia) which has been previously used for bathymetric SLAM [22], while the second one is a full 3D dataset gathered in the Sant Feliu de Guíxols Harbor (Spain) using the *Girona 500* AUV [36] with the multibeam mounted on a pan and tilt unit. The parameters and thresholds that were set for the execution of the algorithm in these experiments can be found in Table 1. Unfortunately, none of the datasets used during the experimental testing have ground truth of the terrain. Therefore, the only option to assess the performance of the algorithm is evaluating the consistency of the resulting map.

### 5.1. Bathymetric Survey

This dataset includes depth from a pressure sensor, bottom lock velocities from a DVL, attitude measurements from an AHRS and bathymetric data from a multibeam echosounder installed in the conventional down-looking configuration. The mission surveyed a rectangular area of geological interest several times to generate multiple loop closures. The explored area, mainly flat, has a number of pockmarks with depths of approximately three meters.

**Figure 8:**
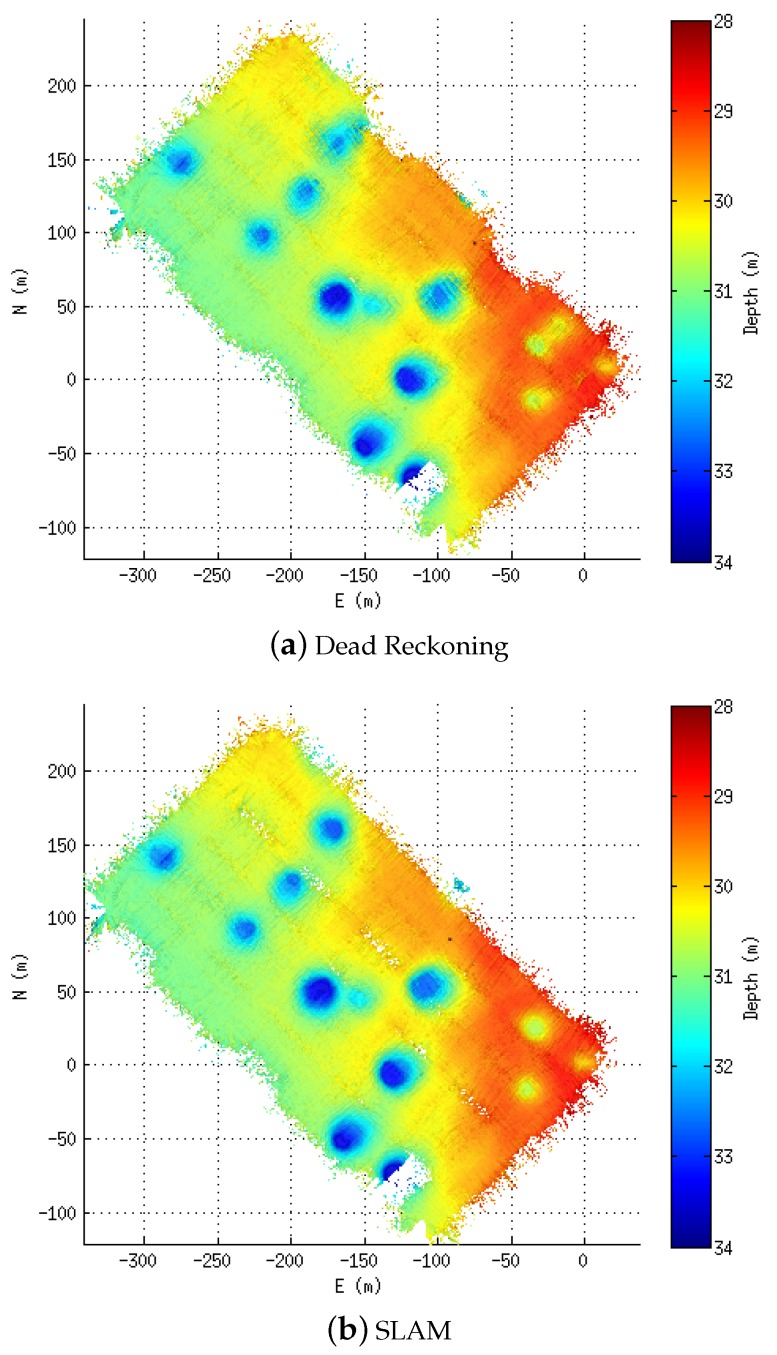
Bathymetric maps of the area. The color goes from deep (**dark blue**) to shallow (**dark red**). The bathymetry is gridded at 0.5 m. (**a**) Dead Reckoning; (**b**) SLAM.

Figure 8 shows the elevation maps built using the dead reckoning navigation (Figure 8a) as well as the one obtained with the proposed technique (Figure 8b). In these two maps, it is possible to observe several differences in the pockmarks. While in the corrected solution (Figure 8b), the pockmarks appear clearly and without much bathymetry-related artefacts on the dead reckoning map, and they are blurred and with some artefacts.

**Figure 9:**
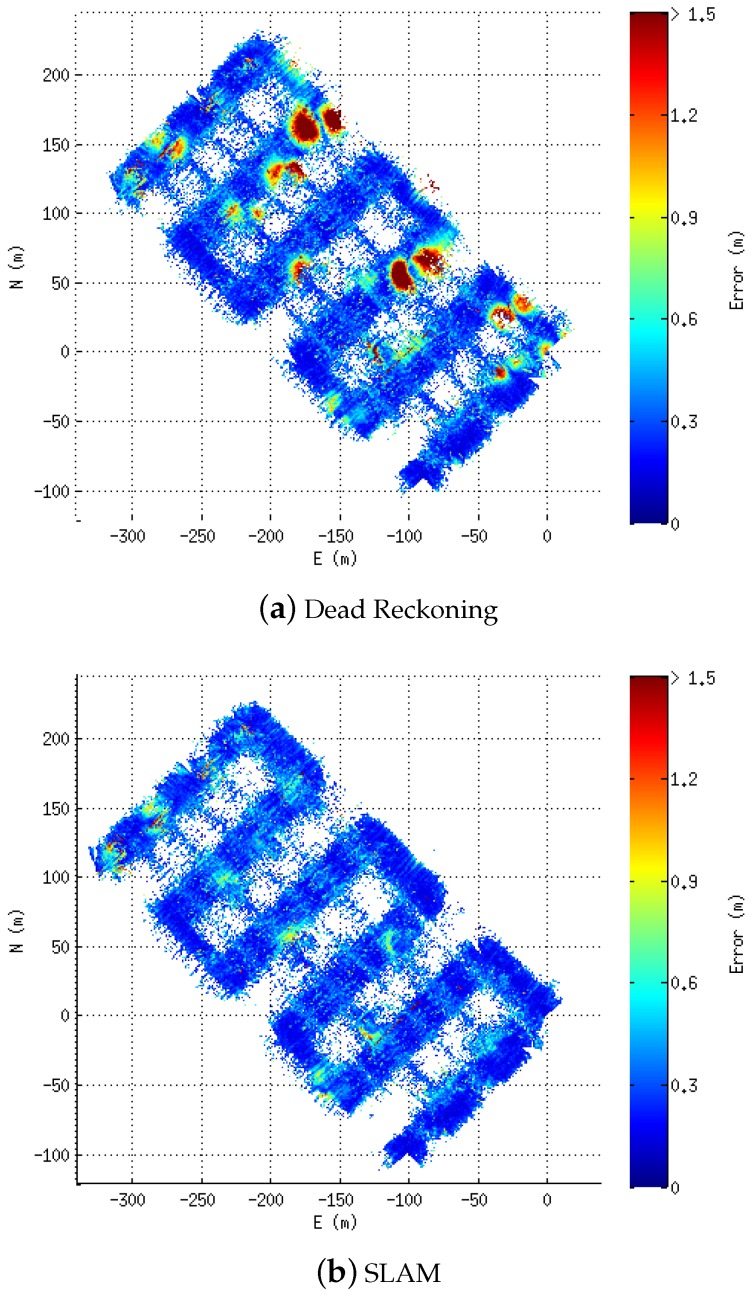
Consistency-based error maps. The error is color plotted from low (**dark blue**) to high (**dark red**) with 0.5 m grid resolution. (**a**) Dead Reckoning; (**b**) SLAM.

To better assess the correction, the consistency-based error [37] is computed for each cell of the bathymetric map. In Figure 9, it can be seen how the areas of high discrepancy (yellow to dark red) on the dead reckoning error map (Figure 9a) are drastically reduced when the proposed technique is applied (Figure 9b). Table 2 contains the numerical evaluation of the results over the bathymetric data. There, it is possible to see that using the 2.5D statistics (Sum and Mean for the consistency-based error) the improvement is around 19%. Moreover, an additional 3D statistic we have named #Cells has been computed. This statistic consists in counting the number of cells that each map occupies within the same 3D grid. If a map occupies less cells, it is probably because their point clouds are more densely packed due to a better registration. Using this statistic, the improvement of the proposed approach compared to the dead reckoning navigation is 2.17%.

### 5.2. 3D Experiments

**Figure 10:**
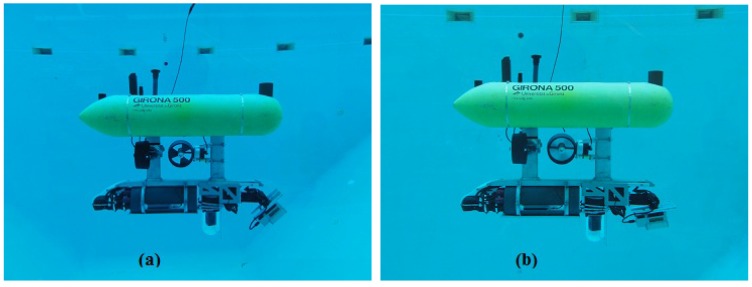
The *Girona 500* AUV in the water tank with the configuration used for the experiments. The multibeam sonar and the pan and tilt unit can be seen at the lower-right side of the vehicle facing in two different directions. In (**a**) the multibeam is tilted at a pitch of around 45° while in (**b**) it is in a downward-looking position.

**Figure 11:**
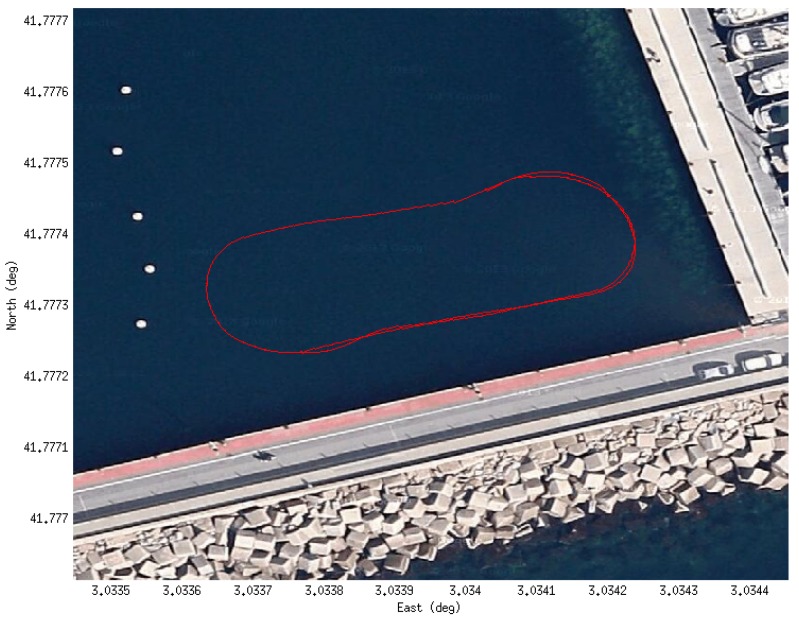
Trajectory of the experiment over the Google Maps image of the St. Feliu de Guíxols harbor.

The data was gathered during some field trials for the MORPH European project in 2015. The experiment involved a formation of four marine vehicles (an Autonomous Surface Craft (ASC) and 3 AUVs) exploring a submerged area of the St. Feliu harbor. The *Girona 500* was leading the formation while exploring with the multibeam sonar mounted on a pan and tilt unit (Figure 10) both the seabed and the pier walls, so the formation could be adapted to the presence of obstacles. The mission performed one and a half loops following a zero-shaped trajectory at one corner of the harbor (Figure 11).

During the experiment, the *Girona 500* (Figure 10) was equipped with a DVL, an AHRS, a pressure sensor and a multibeam echosounder. An acoustic modem on *Girona 500* was also used to gather position measurements from a USBL mounted on the ASC navigating on the surface with help of a GPS receiver. The multibeam mounted on the pan and tilt unit allowed us to get full 3D scans by vertically steering the multibeam in front of the robot. Note that, in this experiment, the closure of the surface patches is determined by the completion of a sweep of the pan and tilt, and not by the size or richness of the covered area (see Table 1).

As far as we know, there is no general method to evaluate the consistency of a 3D map. However, it is possible to use the 3D statistic #Cells presented in the previous section. As previously commented, if a map occupies fewer cells, it is probably because their point clouds are better registered. Nonetheless, this has to be supervised since it is also possible to find other positions of the point clouds that can minimize the number of occupied cells. For this reason, the consistency of the 3D experiments will also be evaluated subjectively (visually assessing the consistency) as well as numerically (counting the number of occupied cells).

**Figure 12:**
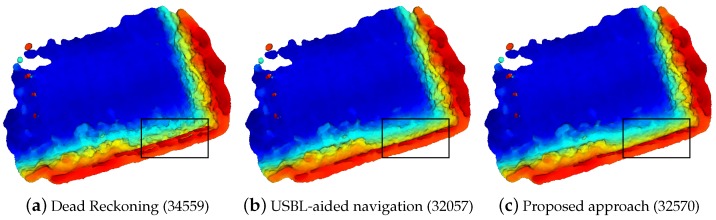
Top view of the 3D reconstruction of St. Feliu Harbor using dead reckoning navigation (**a**), usbl-aided navigation (**b**) and the proposed SLAM algorithm (**c**).The bottom part of the model is the vertical wall of the pier. Under each view, written inside parentheses, the number of cells occupied by each model’s point clouds can be observed. The meshes are reconstructed using [38] and colored according to the depth (deeper parts are in blue, shallower ones in red).

**Figure 13:**
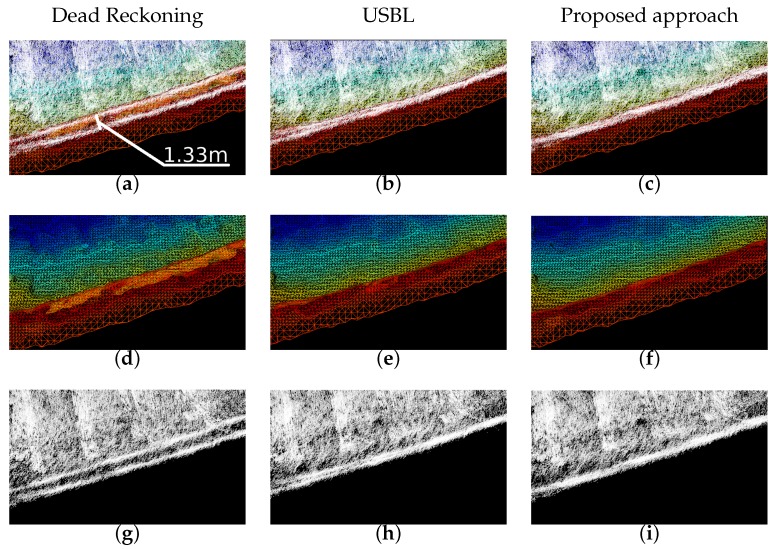
Zoom in the highlighted area of Figure 12. First row (**a**-**c**) shows the point clouds and the reconstructed meshes. Second (**d**–**f**) and third (**g**–**i**) rows show the mesh and point clouds respectively. The columns, from left to right are related to the results obtained with: (1) dead reckoning (**a**, **d** and **g**); (2) USBL-aided (**b**, **e** and **h**) and (3) proposed approach (**c**, **f** and **i**).

The top view of the 3D maps produced after the experiments are presented in Figure 12. There, three surfaces are created for different navigation methods: dead reckoning (Figure 12a), USBL-aided (Figure 12b) and the currently proposed algorithm (Figure 12c). Regarding the number of occupied cells, the proposed method occupies 32570 cells, 5.76% less than the dead reckoning model (34559) while the one aided by the USBL occupies 7.24% less cells (32057). Moreover, the black squares represented in each one of the views highlight the places where it is easier to observe the consistency of the map near to the harbor wall. This area is analyzed in detail in Figure 13. In the left column, the one corresponding to the dead reckoning navigation (views Figure 13a,d,g), clearly shows two parallel lines on the point clouds which correspond to the wall being observed during the first and second laps of the mission. In the other two columns, the one corresponding to the usbl navigation (views Figure 13b,e,h), and the one of the proposed SLAM algorithm (views Figure 13c,f,i) show a single wall, and, thus, a better agreement between the different scans. However, if the point cloud from the USBL navigation is analyzed in detail in the bottom left corner (see Figure 13h), there are still some residues of the two observations of the wall that do not appear in the one from the proposed approach.

## 6. Conclusions

This paper has presented a probabilistic underwater 3D SLAM for multibeam sonar data that deals with the subdivision of the surface into patches, taking into account the motion uncertainty during their formation. An adaptive sampling procedure for the sensor data has been introduced to deal with areas of the patches that are not relevant (*i.e.*, without relief) to avoid the pICP converging to local minima as well as reducing the computational time. Furthermore, an heuristic has been used to decrease the complexity of the association step of the pICP from $O(n^{2})$ to O(n) taking advantage of the probabilistic ellipsoid of each point and using a support grid.

The algorithm has been tested using two real world datasets. In both of them, it is possible to observe how the consistency of the model obtained using the proposed algorithm is higher than that obtained with dead reckoning and is even comparable to the one obtained using USBL navigation in the case of the 3D dataset.

Future work will have to focus on correcting the internal patch error. In the method presented here, only the relative positions of the patches are corrected, but the patch itself is not modified once closed. Although the proposed method has been proved to be useful for obtaining consistent maps, it is not possible to use it online due to its computational cost if the point sampling is not tuned properly. Therefore, further investigation could be done in this field to allow the algorithm to work online. Finally, in the future, we plan to test the algorithm using a camera-laser system, which produces data of similar characteristics to that of a multibeam sonar (2D swath profiles) but with a much different uncertainty level in the measurements.

## Figures and Tables

**Table 1:** Thresholds used for the experiments.

	Experiment	
	2.5D	3D
Minimum patch size (Section 2.2)	30 m	-
Maximum patch size (Section 2.2)	80 m	-
Normal occupancy (Section 2.2)	23%	-
Patch overlapping (Section 4.2)	30%	30%
Point cloud subsampling (Section 3.4)	0.5 m	1.5 m
Relative displacement to switch from point-to-point to point-to-plane association (Section 3)	1 cm	1 cm

**Table 2:** Numerical results of the algorithm applied to the pockmarks dataset. The first column (Sum) contains the sum of the error in all the cells, the second one (Mean) contains the mean of the error while the 3rd one (#Cells) contains the number of cells occupied on a 3D grid of 0.5 m resolution.

	Sum	Mean	#Cells
Dead reckoning	70,986.2	0.3988	37,3121
SLAM	57,521.8	0.3223	36,5014
Improvement *	18.97%	19.2%	2.17%

* The improvement is computed as $\frac{dr-slam}{dr}$ where dr stands for dead reckoning.
